# Virtual reality interventions designed to support parents during and throughout the first year after birth: A scoping review

**DOI:** 10.1177/20552076241245373

**Published:** 2024-04-22

**Authors:** Victoria Fallon, Sian M Davies, Sergio Silverio, Lisa Creagh

**Affiliations:** 1 Department of Psychology, Institute of Population Health, Faculty of Health and Life Sciences, University of Liverpool, Liverpool, UK; 2 School of Psychology, Faculty of Health, Liverpool John Moores University, Liverpool, UK; 3 School of Life Course and Population Sciences, Faculty of Life Sciences and Medicine, London, UK; 4 Lisa Creagh Limited t/a The Holding Time Project, Plus Accounting, Brighton, East Sussex, UK

**Keywords:** Virtual reality, scoping review, postpartum, interventions, parents, childbirth

## Abstract

**Objective:**

Virtual reality (VR) has become increasingly popular in clinical and health settings where it has been used for a wide range of purposes. A recent scoping review explored VR applications to assist pregnant women and found that VR was a useful method to be used for a range of different purposes in both pregnancy and labour. However, no such review exists for the period after birth.

**Method:**

We aimed to search for studies that used VR to support parents during birth and in the first year postpartum (Population) in different settings (Context), and finally provided data on the characteristics, reported effectiveness and experience of VR interventions (Concept). Two hundred and fifty-one studies were identified, of which ten were eligible. Two authors independently extracted data including study design, participants and results.

**Results:**

Findings indicate that VR has been used effectively in this context to alleviate depression anxiety, and multiple domains of pain and to improve childbirth satisfaction. The majority of the studies explored the use of VR technology on outcomes such as pain and anxiety during labour and birth. The studies included used a broad range of VR hardware and software. All of the studies reported positive experiences of using VR.

**Conclusions:**

Across these studies, VR was found to be effective in terms of both physiological and psychological outcomes. There are many unexplored maternal and infant focused applications of VR which warrant further investigation as emerging evidence indicates this is becoming an increasingly accessible method to improve maternal and infant health outcomes from pregnancy through to parenthood.

## Introduction

Virtual reality (VR) is a computer-based technology that has seen an exponential surge in popularity due to increased affordability.^1^ There are three primary categories of VR simulations used today: non-immersive, semi-immersive and fully-immersive simulations.^2^ Non-immersive VR uses a computer or video game system and users interact with the virtual environment (VE) with devices such as a mouse or a joystick. Semi-immersive VR provides users with a partially VE simulating via 3D graphics. Immersive VR uses VR glasses or a head-mounted display providing the user with high-resolution content and a wide field of view. The VE is the simulated scenario generated by the computer or device which is designed to be explored, so users can interact with their environment.^3^ In multi-user or collaborative virtual environments two or more users can share the same simulation and communicate and/or interact inside it.^3^ Augmented reality is a newer technology which combines adding virtual objects into real life, in real time.^4^

VR provides opportunities to create and recreate simulated environments ‘where the testing, training, teaching, and treatment of cognitive, emotional, and sensorimotor processes can take place under stimulus conditions that are not easily deliverable and controllable in the physical world’.^5^ As a result of this, VR technologies are increasingly used in different settings including sports,^6^ the arts,^7^ the military,^8^ industry,^9^ education,^10^ entertainment^11^ and healthcare.^12^ VR has become increasingly popular in clinical and health settings^1^ where it has been used for a wide range of purposes including medical simulation,^13^ remote and live surgery,^14^ training health care professionals,^15^ facilitating pain management,^16^ improving psychological health^17^ and exercise rehabilitation.^18^

A recent scoping review explored VR applications to assist pregnant women and found nine studies which demonstrated that VR was a useful method to be used for a range of different purposes in both pregnancy and labour.^19^ Four studies had used VR technology to reduce the anxiety of pregnant women, four studies had applied VR for decreasing pain during labour, and one study used VR to support physical activity in pregnancy. However, no such review exists for the period after birth which makes this an important and necessary undertaking to add to the literature base on utilisation of VR in maternal health settings. The postnatal period is a complex period in a woman's life with a multitude of unique biopsychosocial transitions during which VR may have the potential to support. Furthermore, the prenatal review used a small number of databases (*n* = 3), search terms regarding labour and birth were limited, and the search strategy was restricted (keywords were searched only in the Titles/Abstract fields). The authors acknowledge this may have lacked sensitivity and omitted relevant work. While our focus was on the postnatal period, we deemed it relevant to include studies relating to birth and delivery and overcome the limitations of the previous review. Therefore, this scoping review has the research question of ‘What are the characteristics, reported effectiveness, and experiences of VR interventions designed to support parents during and throughout the first year after birth?’ It therefore aims to synthesise the existing literature about the characteristics, reported effectiveness and experience of VR interventions designed to support parents during and throughout the first year after birth.

## Method

A protocol was developed using the scoping review methodological framework proposed by Arksey and O’Malley^20^ and further refined by the Joanna Briggs Institute.^21^ The draft protocol received feedback from the research team, including methodologists and a creative artist involved in VR intervention development. The final protocol is available upon request from the corresponding author. The PRISMA extension of scoping reviews (PRISMA-ScR) checklist was used to report information throughout the article.

### Eligibility criteria

Published studies were included if they reported data on the reported effectiveness and/or experience of VR interventions designed to support parents during birth and in the first year postpartum. This included all interventional, observational and qualitative study designs that provided information on outcomes (e.g. reduction in anxiety) and/or experiences (e.g. satisfaction with the intervention). Any outcome or experience were considered eligible. All types of VR were considered including non-immersive VR, fully-immersive VR, semi-immersive VR, augmented reality and collaborative VR. Studies that did not use VR as an intervention to support birth or the first year of life, that used VR to train healthcare professionals, that used VR for parents with children > 1, or that focused on the infant rather than the parent, were excluded. We further excluded study protocols, reviews, books, periodicals, and articles with a full text in a language other than English.

### Information sources

Databases searched included: The Cumulative Index of Nursing and Allied Health Literature (CINAHL), MEDLINE (Ovid), PsycArticles, Embase, Web of Science and Global Index Medicus. Additional methods involved author runs, backward and forward chaining. The population, concept, and context (PCC) structure was used to identify search terms. We aimed to search for studies that used VR to support parents during birth and in the first year postpartum (Population) in different settings (Context), and finally provided data on the reported effectiveness and/or experience of using VR technology (Concept). Search terms were refined following a priori scoping exercises and modified for specific databases as appropriate. Keywords included: ‘virtual reality’, ‘VR’, ‘virtual intervention’, ‘augmented reality’ and ‘labor’, ‘labour’, ‘birth’, ‘postnatal’, ‘postpartum’, ‘maternal’, ‘mother’, ‘parent*’. A copy of the full electronic search strategy is available upon request. A manual search of reference lists of included studies and relevant reviews was also conducted.

### Study selection

A three-stage screening protocol was followed. Titles were first assessed and any articles that were evidently unsuitable were excluded at this stage. Abstracts were then screened and excluded where appropriate with written justification. Finally, the full text of each eligible article was read thoroughly by two authors (VF and SMD) to determine inclusion in the review.

### Data extraction

Two review authors (VF and SMD) extracted data from the included studies. Any inconsistencies were resolved by discussion or, where necessary, LC was consulted. For each study, general characteristics extracted included authors, year of publication, country, study design, sample size and characteristics, study objective. Specific characteristics of the intervention were also extracted including VR components (software and hardware), data collection, results and pertinent methodological details. Where necessary, authors were contacted to identify/confirm any missing or ambiguous data.

### Data analysis

Descriptive summaries were provided for general study characteristics and characteristics of the VR hardware and software. Results were then synthesised and reported based on physical and psychological outcomes. A summary of the experiences of those using VR was also provided.

## Results

**Figure 1.**
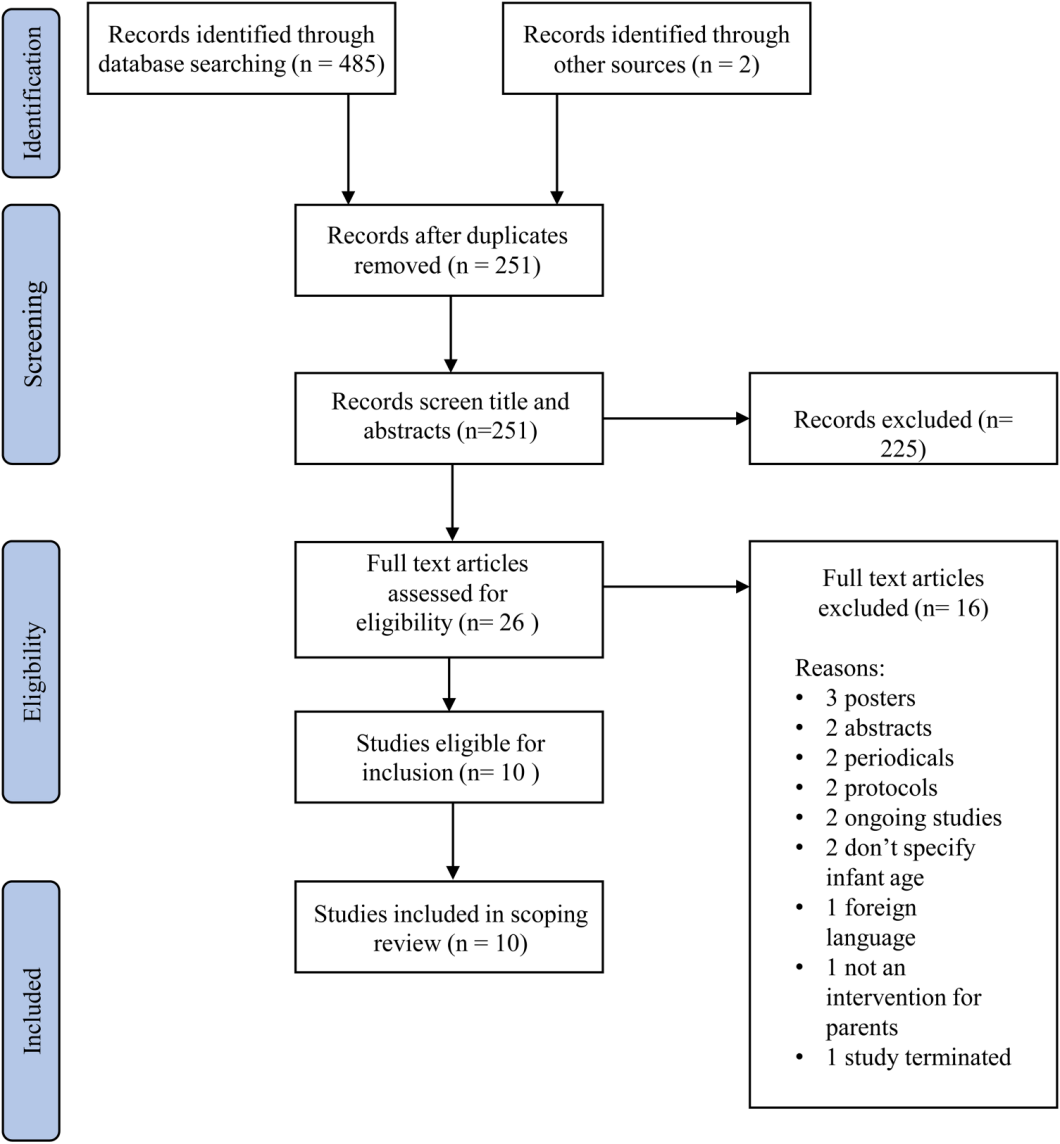
PRISMA scoping review flowchart.

Following searches using CINAHL, MEDLINE (Ovid), PsycArticles, Embase, Web of Science and Global Index Medicus, 485 studies were identified. Two additional studies were identified via reference list searches. Two-hundred fifty-one studies were remaining after 234 duplicates were removed. These studies then underwent title and abstract screening. Twenty-six full text articles remained and were reviewed for eligibility resulting in 10 studies meeting the inclusion criteria for this study (see Figure 1).

### General study characteristics

Ten articles met inclusion criteria and were included in this review.^22–31^ The majority of the studies (*n* = 8) were published between 2019 and 2021^22–25,27,28,30,31^ with the exception of one study published in 2015^26^ and one published in 2016.^29^ Four of the studies were conducted in Iran,^23,26,27,29^ two in Turkey,^22,25^ and one in the US,^24^ Mexico,^28^ New Zealand^30^ and China.^31^ Nine of the included studies were randomised control trials^22–29,31^ and one study was a single case-study trial.^30^ A total of 726 participants were included in the 10 studies, with sample size ranging from 4^28^ to 273.^25^ Two studies aimed to reduce pain^25,26^ and one study aimed to reduce anxiety.^29^ Some studies (*n* = 5) reported multiple outcomes (e.g. pain and anxiety)^22,24,27,28,31^ and one study reporting both depression and anxiety.^30^ One study examined childbirth satisfaction.^23^ The majority of the studies (*n* = 7) explored the use of VR technology on outcomes such as pain and anxiety during labour and birth.^22–25,27,28,31^ Five studies were identified that examined VR during labour that were not identified in the previous prenatal scoping review.^22,23,25,27,31^ Two studies investigated the use of VR technology following birth during episiotomy^26,29^ and one study examined the role of VR technology postnatally (see Table 1).^29^ All of the studies used immersive VR.

**Table 1.** General characteristics of the included studies.

**First author's name**	**Year of publication**	**Country**	**Type of study**	**Sample size**	**Study objective**
Akin, et al.	2021	Turkey	RCT	100	To determine the effect of showing images of the foetus to the pregnant women with the virtual reality glass during labour process on labour pain, childbirth perception and anxiety level
Ebrahimian, et al.	2021	Iran	RCT	93	To compare the effects of watching virtual reality videos and chewing gum on the length of delivery stages and maternal satisfaction
Frey et al.	2019	US	Pilot RCT	27	To investigate the use of VR on pain and anxiety during unmedicated labour
Gur, et al.	2020	Turkey	RCT	273	To investigate the effects of cognitive behavioural techniques using virtual reality on birth pain
Jahani, et al.	2015	Iran	RCT	30	To determine the effect of using video glasses on pain reduction in primiparity women during episiotomy repair
Momenyan, et al.	2021	Iran	RCT	52	To assess the impact of immersive VR analgesia on cognitive, affective and sensory components of pain, anxiety and nausea in nulliparous women experiencing un-medicated labour
Mosso, et al.	2019	Mexico	RCT	4	The objective is to reduce stress and anxiety before newborn delivery in order to reduce pain and other complications during the surgical procedure
Shourab, et al.	2016	Iran	RCT	30	To determine the effect of audio-visual distraction (VR) on anxiety in primiparous women during episiotomy repair
Stamou, et al.	2021	New Zealand	Single-case study trial	15	To investigate the clinical usefulness of combining CBT with virtual reality for the treatment of postnatal depression
Wu, et al.	2020	China	RCT	102	To explore the effect of VR technology on the anxiety state of parturient in labour analgesia

### Characteristics of the VR

The studies included used a range of VR hardware used in varying combinations. Three studies implemented the VR technology via a smartphone^22,24,25^ and three studies implemented the VR technology via a computer or DVD player.^26,29,30^ Five studies utilised a head mounted display participants wore^24,27,28,30,31^ and four studies provided VR glasses/goggles for participants to wear.^23,25,26,29^ One study additionally provided a joystick for participants to use during the VR program.^30^ Similarly, a range of VR software was employed, Akin et al.^22^ displayed foetal ultrasound images taken antenatally to participants. Ebrahminian at al.^23^ presented 360° views of nature scenes. Frey et al.^24^ displayed simulations of ocean scenes and animals alongside relaxing music. Gur et al.^25^ used VR to show participants digital photographs of content infants accompanied by classical music. Jahani et al.^26^ provided participants with a 3D film of ocean animals. Momenyan et al.^27^ displayed 360° videos of peaceful landscapes. Mosso et al.^28^ provided participants with the option to select one of three VR nature scenarios. Shourab et al.^29^ displayed VR videos of ocean animals. Stamou et al.^30^ provided participants with a virtual household environment to navigate containing various stressors. Wu at al.^31^ showed participants VR programs such as ocean scenes (see Table 2).

**Table 2.** Intervention characteristics of the included studies.

**First author's name**	**Timing of intervention**	**VR components**	**Data collection method**	**Results**	**Experience**
Akin, et al.	During labour	**Hardware:** Smartphone, VR Box 3D virtual reality glass **Software:** App on maternal phone displaying foetal ultrasound images taken antenatally (28 weeks).	Visual Analogue Scale (VAS) for measuring pain at 9 cm dilation; Perception for the Scale of Supportive Care Given During Labor (POBS) and Perinatal Anxiety Screening Scale (PASS) 2 h after labour.	Physical: The perceived pain level when cervical dilatation was 9 cm was significantly lower in the intervention group (*p* < 0.05). Psychological: The POBS total scores were significantly higher and anxiety scale total mean scores were significantly lower (*p* < 0.001) in the intervention group.	When asked about their thoughts on labour after the labour process, women who did not use the VR stated that the act of giving childbirth was more painful and scary compared to those who used the VR.
Ebrahimian, et al.	During labour	**Hardware:** VR goggles **Software:** 360° view of rivers, shores, waterfalls, and lakes.	The Mackey Childbirth Satisfaction Rating Scale was completed by all participants after their postpartum condition became stable.	Physical: There was no significant difference in the mean length of the active and second phases of parturition between the two intervention groups, but this value in the intervention groups (active parturition VR mean = 271.12, SD = 52.05; second stage VR mean = 21.61, SD = 6.50) was significantly lower than that of the control group (active parturition mean = 304.51, SD = 65.66; second stage mean = 26.45, SD = 6.60). Psychological: There was no significant difference between the mean maternal childbirth satisfaction scores in the two intervention groups of virtual reality and chewing gum (*p* = 0.339). However, the mean score in the intervention groups was higher than that of the control group (*p* < 0.001).	Childbirth satisfaction was the primary outcome, no other experience data was collected.
Frey, et al.	During labour	**Hardware:** a Samsung GearVR head-mounted display powered by a Galaxy S7 phone, a hand control, and noise-reducing headphones powered by a parallel S5 phone **Software:** curious manatees from the Ocean Rift, scuba diving simulation with sounds of manatee calls and breathing underwater, on with sounds of manatee calls, and breathing underwater, additional relaxing music was supplied from night-time sleep by Brain.fm.	Ratings of 3 separate pain outcomes using numeric rating scale (NRS) tools: amount of time spent thinking about pain (cognitive pain dimension), pain unpleasantness (affective pain dimension), and worst pain intensity (sensory pain dimension) they experienced.	Physical: The NRS scores for worst pain intensity (sensory pain) were significantly lower in the VR condition (SE −1.5 [95% CI, −0.8 to −2.2] and SMD −0.8). The NRS scores for affective pain (SE −2.5 [95% CI, −1.6 to −3.3] and SMD −1.0), cognitive pain (SE −3.1 [95% CI, −2.4 to −3.8], and SMD −1.7) were also significantly lower in the VR condition. Psychological: The NRS scores for anxiety were also lower in the VR than non-VR condition (SE −1.5 [95% CI, −0.8 to −2.3] and SMD −0.7).	82% reported very much/completely enjoying VR use during labour. 70% of participants would be very/completely interested in new VR development specifically for childbirth.
Gur, et al.	During labour	**Hardware:** Samsung Gear VR2 Virtual Reality Glasses and smartphone. **Software:** Digital photograph album composed of photographs of healthy, not crying, and calm newborns, videos of newborns accompanied by classical music, and an introductory film of Turkey.	Visual Analogue Scale (VAS): In this scale, the participants marked the severity of their pain on a 10 cm ruler with one end stating ‘no pain’ and the other end ‘worst possible pain’. Verbal Rating Scale (VRS): This scale is based on participants’ choice of word that defines their pain state. The following words to define the severity of pain were: mild (1), uncomfortable (2), intense (3), very intense (4), and unbearable (5). Participants were asked to select the most suitable category for them.	Physical: The difference between participants’ intergroup mean VAS and VRS pre-test scores was not statistically significant (*p* = 0.08, *p* = 0.09). However, the difference between participants’ intergroup mean VAS and VRS post-test scores was statistically significant (*p* < 0.001, *p* = 0.02).	Participants reported that this experience helped them to relax, increase their control, focus on their baby, reduce anxiety, and would recommend the use of VR during labour.
Jahani, et al.	After birth – during episiotomy repair	**Hardware:** The VR equipment consisted of a video player (3D Blu-ray/DVD player full HD model connected to a pair of video glasses (Wrap 920 system) including the connection cables. Video glasses include two miniature LCD viewing screens (for the right and left eyes) and two external headphones. The unit included an external remote control device. **Software:** 3D film (IMAX Dolphin and Whales 3D).	The pain intensity was measured based on a Numeric Pain Rating Scale (0-100) before and during the four stages of repair, namely the first minutes of infiltration anaesthesia during Hyman repair, during skin repair, immediately after the repair and the first hour after the repair. Data was collected using a questionnaire (including demographics, labour and delivery segments) and the VR equipment. After the episiotomy repair, the time was recorded from the first suture to the last. In addition, parturient satisfaction was recorded before and after episiotomy repair.	Physical: The result showed significant differences on episiotomy incision depths between the intervention group and the group receiving standard care (*U* = 69.0, *p* = 0.04). Results showed patient with a VR distraction condition reported a significantly lower repair time than patients in non-VR group. Additionally, a significant difference was found between the groups, based group effect (*p* = 0.04) and different stages (*p* < 0.0001). The pattern of findings, as indicated by ‘group multiplied by the different stages of interaction effect’, was statistically significant for the pain intensity (group and stages *p* = 0.04). Severe pain (from 80 to 100) was reported in 60% of the VR group and 20% of the non-VR group. Only 6.7% of the VR group and 26.7% of the non-VR group had severe pain on Hyman repair stage.	No experience data was recorded.
Momenyan, et al.	During labour	**Hardware:** Head mounted display powered by a Samsung S3, and a noise reduction headphone**.** Head movement was tracked using the Samsung S3's inertial measurement unit (IMU) sensor. **Software:** 360 degrees video of nature containing beach and peaceful landscape shown along with the sound of nature. To present this video, an Android application was developed using the Google VR SDK ** **	Numerical rating scale in the first and second stage of labour to assess 3 separate pain outcomes, anxiety and nausea. Patients rated based on the amount of time they spent thinking about pain (cognitive pain dimension), pain unpleasantness (affective pain dimension), worst pain intensity (sensory pain dimension), the amount of anxiety, and the amount of nausea they experienced.	Physical: The mean score of cognitive pain was significantly lower among the intervention group in comparison to the control group in the first stage (*p* = 0.01) but not in the second stage (*p* = 0.55). The mean score of affective pain was not statistically significant between the two groups during the first and the second stage. Within the first stage, the mean score of sensory pain in the control group and the intervention group was significant (*p* = 0.03), but it was not significant in the second stage (*p* = 0.06). Psychological: There was a significant difference in anxiety between the two groups in the two stages. The mean of anxiety was significantly higher in the control group in comparison to the intervention Group (*p* = 0.04 stage 1; *p* = 0.01 stage 2). There was not a significant difference between the two groups in terms of nausea during the first stage (*p* = 0.09) and the second stage (*p* = 0.54).	No experience data reported
Mosso, et al.	During caesarean delivery	**Hardware:** Head mounted display (HMD) linked to a laptop with game pad to navigate the VR world. **Software:** There are 3 VR scenarios (Enchanted Forest, Cliff, and Castle) that the mother can choose from	A visual analog scale (VAS) was used to measure pain and anxiety (0 = no pain/anxiety, 10 = high pain/anxiety) before, during, and after caesarean delivery.	Physical: Pain during caesarean delivery under epidural analgesia decreased 91.89% aided with VR. In the control group pain increased 61%. Psychological: Overall, all patients experienced reduction in stress and anxiety using VR.	No experience data reported.
Shourab, et al.	After birth – during episiotomy repair	**Hardware:** Glasses (Wrap 920) with two headphones manufactured by Vuzix; stereosound with frequency of 60 Hz; resolution 280 × 640 pixels; virtual image 67 in at a distance of 3 m, 31° angle; 85 g weight with the support of formats of multianaglyph and side by side. A DVD Player/3D Blue-Ray Player, Full HD 1080p picture performance; model BD660 made in Indonesia with the connection cable and composite input **Software:** IMAX Dolphin and Whales 1080p Half-SBS AC3	Rating scale of anxiety (0–10 scale) of the parturient women before the repair and during the Hyman repair. 15 min after the end of the episiotomy repair each participant completed the STAI	Psychological: Anxiety scores were not significantly different between the two groups (wearing video-glasses and receiving routine care), but anxiety scores were lower in the intervention group during and after repair (*p* < 0.001).	All mothers were satisfied with their pregnancy and baby's gender.
Stamou, et al.	Postnatal period	**Hardware:** Windows 7, Dell OptiPlex 3020 PC (Intel Core TM i5-4670@3.40 GHz, RAM 8 GB), LCD screen (Dell E1910C, 19″, 1440 × 900), Logitech HD Webcam C270, Tritton Kunai Stereo Headset, and Logitech X3D for a joystick. The mouse was used by the therapist, while the joystick is used by the participants to navigate themselves in the virtual environment. **Software:** Audio and video communication between the two computers used ‘Video Chat’ developed by Midnight Status. The virtual environment depicts a middle-class house, with two bedrooms, a bathroom, a kitchen (dining room), and a living room, all with suitable furniture such as beds, sofas, drawers, curtains, kitchen appliances, and bathroom utilities. There is also an outside area with a garden and a playground. The stressors of the VR programme were divided into three main categories, home stressors, the toddler's stressors and the neighbour stressors.	EPDS, GAD-7 and Kessler-10 at pre-treatment, post-treatment, and follow-up (3 months post-intervention). Daily questionnaire (6 items around mood) completed daily during all phases of study	Psychological: Kessler-10: baseline vs post-treatment Cohens D = 0.67 (medium); post-treatment vs follow-up = 1.02 (large) GAD-7 baseline vs post-treatment 1.06 (large); post-treatment vs follow-up = 1.47 (large) EPDS baseline vs post-treatment 1.17 (large); post-treatment vs follow-up = 0.83 (large). Daily questionnaire showed clear improvement from session to session, starting from baseline until the follow-up periods in the participants from the three different baselines established. However, the improvement in symptoms becomes clearer following the VR Session.	VR enhanced awareness, decision making, and self-appreciation within the individual and can also have real-life applications. The combination of VR and CBT was feasible, and the use of VR was well accepted.
Wu, et al.	During labour	**Hardware:** VR headsets (HTC VIVE, not in the medical equipment scope) **Software:** VR programs purchased from the official website https://store.steampowered.com/ such as Ocean Rift.	State Trait Anxiety Inventory (state and trait scales) and Numeric Rating Scale for Pain (0-100) collected before and 30 min after labour analgesia/ labour analgesia + VR	Physical: After labour analgesia, the pain scores of the two groups (analgesia/analgesia + VR) were significantly reduced (*p* = 0.000), but the pain of the mothers in the VR group were relieved more significantly (*p* = 0.000). Psychological: The anxiety scores of the two groups (analgesia/analgesia + VR) were significantly reduced (*p* = 0.000), but the anxiety of the mothers in the VR group were relieved more significantly (*p* = 0.000).	VR significantly improved the overall satisfaction of mothers from baseline to follow up (9 vs. 10, *p* = 0.000).

### Reported effectiveness according to physical and psychological outcomes

#### VR and physical outcomes

Seven of the studies included demonstrated the VR intervention groups having a significant analgesic effect on pain during labour^22,24,27,28,31^ and episiotomy repair^26,29^ compared to the non-VR groups.

#### VR and psychological outcomes

Eight studies demonstrated lower anxiety^22,24,27–31^ and depression^30^ scores in the VR intervention groups compared to the control groups. Additionally, one study found that maternal childbirth satisfaction scores were higher in the VR groups in comparison to the control group.^23^

#### Experiences of those using VR

Six studies reported data on the experiences of participants while using the VR.^22,24,25,29–31^ Two of these studies reported quantitative survey data^24,31^ and four reported qualitative data.^22,25,29,30^ Five of the studies reported on VR experience during labour,^22,24,25,29,31^ and one reported on VR experience during combined treatment for postnatal depression.^30^ All of the studies reported positive experiences of using VR. Childbirth was considered less painful in one study^22^ and less anxiety provoking in two studies^22,25^ while using VR. VR aided focus, control, relaxation and satisfaction with childbirth. VR was also well accepted, recommended and enjoyed by women during childbirth in three studies.^24,25,30^

## Discussion

This scoping review was conducted to identify and review the studies that used VR to help mothers during birth and the first postpartum year. Findings indicate that VR has been used effectively during and immediately after birth to alleviate depression anxiety, and multiple domains of pain, and to improve childbirth satisfaction. There was also preliminary evidence in one study that VR was effective in treating symptoms of postnatal depression. Anxiety and fear during childbirth have been consistently linked to a number of adverse objective and subjective birth experiences^32^ and contribute to higher levels of pain experienced during birth.^33^ The relationship between anxiety and pain is cyclical with anxiety increasing perceptions of pain, and the experience of pain exacerbating anxiety.^34^ It is well documented that difficulties during labour are associated with a range of birth complications which have negative implications for health and wellbeing in the postnatal period for both mother and infant.^32^ Therefore, the use of VR has the potential to disrupt the anxiety-pain feedback loop and reduce the risk of longer-term adverse maternal and infant outcomes.

VR was used predominately during labour and birth, but also for episiotomy repair and postnatal mental health. Furthermore, mothers reported positive experiences of using VR across many of the studies which indicates it is an acceptable intervention across diverse settings in the intrapartum and postpartum period. However, there has been very limited use of VR outside of an intrapartum context with only one study in the review exploring its use further into the postnatal period.^30^ There is potential for VR to be applied as a preventative tool to other psychological and social domains of perinatal health such as supporting the mother-infant relationship, infant feeding, routine infant care and perinatal wellbeing. Furthermore, it has yet to be applied to other settings within maternity care such as the operating room, postnatal ward or Neonatal Intensive Care Unit. This is similar to the prenatal review where the vast majority of studies were also conducted during labour.^19^ There are also numerous applications for VR in the antenatal period that have yet to be explored including virtual tours of the labour and delivery suite to prepare women for birth, to alleviate anxiety in the early pregnancy assessment unit and pregnancy monitoring. Alleviating childbirth-related anxiety during the antenatal period may also improve birth experiences and subsequent postnatal health and wellbeing.^32^

There was preliminary evidence in one study that VR was effective in treating symptoms of postnatal depression, although this was in combination with CBT.^30^ Further investigation of the efficacy of VR for supporting perinatal mental health is necessary, and studies should consider other conditions (e.g. anxiety, PTSD, stress, adjustment disorders) and constructs (e.g. resilience, wellbeing, self-efficacy, psychological flexibility). VR should be explored as both as a standalone tool, and in combination with other therapies, to fully understand its potential. There is evidence for the efficacy of VR for supporting mental health in general adult populations with a recent systematic review reporting that VR was an effective treatment for a range of mental health conditions.^35^ However, while a meta-analysis found that VR-based interventions were more effective than control conditions, they did not perform better than other therapeutic interventions for anxiety and depression.^36^ VR has also been demonstrated to be effective for specific phobias in general adult populations,^37^ which indicates that it may hold potential in a perinatal context for treating maternal phobias, obsessive-compulsive disorders and breastfeeding aversion.

Finally, while undertaking screening, we noted there were two study protocols with research both planned and in progress in perinatal populations, which was encouraging. This included a planned RCT exploring the utility of VR for assisting with perinatal loss and grief,^1^ and a study in progress which examined the efficacy of VR for reducing pain and anxiety during labour.^38^ Given the number of studies which investigate the potential of VR during childbirth, there may be scope to meta-analyse this literature and draw more robust conclusions regarding its use in this context.

### Strengths and limitations

The majority of the studies included were randomised control trials which are considered gold standard in terms of design due to limiting bias and therefore providing high-quality evidence. Furthermore, all of the studies included used inferential statistics which allows generalisability of findings. However, there was heterogeneity across the studies in terms of aims, VR software, outcome measurements and timing of measurement which limits the ability to draw firm conclusions. Furthermore, there were no qualitative studies included in the review specifically exploring the experiences of those using VR during labour or the first year after birth, which is important to consider in future research. Seven of the studies included used generic, pre-designed relaxation software including dolphins, whales, curious manatees, oceans and mountains.^23, 24,26–29,31^ The remaining three studies used bespoke software tailored specifically to the study aims and population.^22,25,30^ While generic software may be cost-effective, it may lack specificity to childbearing populations, therefore co-design of future VR software with experts by experience would be a good method to improve acceptability. In terms of real-world application of VR in this context, considering safety in postnatal populations who may be caring for a baby is also of importance. The studies included in this review used immersive VR with mounted headsets which limits the ability to carry out routine tasks while using the software. Future studies should also examine the ecological validity of using VR in this context, to see whether it translates into the natural maternal environment. It is important to note that scoping reviews can only provide information on the reported effectiveness, but cannot determine actual effectiveness, of interventions. Future research should consider a full systematic review with meta-analysis to establish actual effectiveness.

## Conclusion

Our scoping review found that VR technology has been used during childbirth and the postnatal period in a range of ways, including reduction of pain, anxiety, and stress in labour, and coping with the symptoms of postnatal depression. Across these studies, VR was found to be effective in terms of both physiological and psychological outcomes. Furthermore, mothers reported positive experiences of using VR which indicates acceptability in this population. There are many unexplored maternal and infant focused applications of VR which warrant further investigation as emerging evidence indicates this is becoming an increasingly accessible method to improve maternal and infant health outcomes from pregnancy through to parenthood.

## Abbreviations

DVD: digital versatile disc
PCC: population, concept and context
PRISMA-ScR: preferred reporting items for systematic reviews and meta-analyses – scoping review extension
RCT: randomised controlled trial
STAI: state-trait anxiety inventory
US: United States
VAS: visual analogue scale
VR: virtual reality.
